# Supplementary material for: Phosphorylation of CENP-A on serine 7 does not control centromere function
Source: Nat Commun. 2019 Jan 11;10:175. doi: 10.1038/s41467-018-08073-1 (PMC6329807; doi:10.1038/s41467-018-08073-1)
Supplement: Supplementary file 1 — Supplementary Information [file 41467_2018_8073_MOESM1_ESM.pdf]

SUPPLEMENTARY INFORMATION

Phosphorylation of CENP-A on serine 7 does not control centromere function

Barra et al

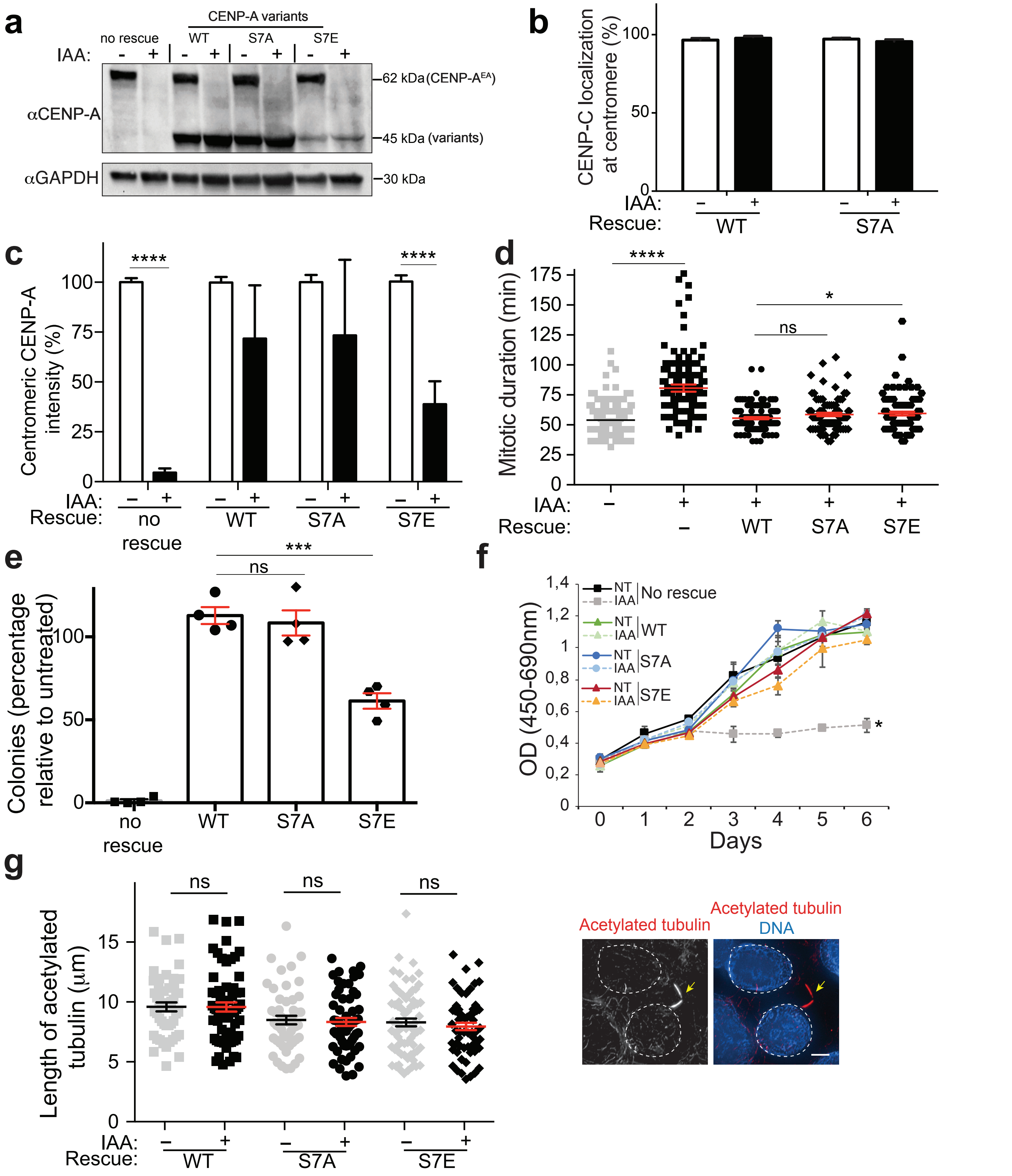

Supplementary Figure 1. CENP-A phosphorylation on serine 7 is not required for short term centromere function (**a**) Immunoblots of cell extracts with antibodies against CENP-A to determine the expression levels of exogenous constructs, and the absence of the endogenous CENP-A protein in the IAA–treated cells. Antibodies against GAPDH are used as a loading control. (**b**) Graph shows the percentage of CENP-C at CENP-B-marked centromeres in the indicated cell lines and following IAA treatment. (**c**) Box and Whiskers (10-90 percentile) analysis of CENP-A intensity at centromeric regions in the indicated cell lines. Error bars represent the SEM of three independent experiments. Unpaired t test: **** $p < 0.0001$. (**d**) Quantification of mitosis duration in the indicated cell lines. Each individual point represents a single cell. Time in mitosis was defined as the period from NEBD to chromosome decondensation. Error bars represent the SEM of three independent experiments. Unpaired t test: * $p = 0.027$; **** $p < 0.0001$. (**e**) Bar graph shows the percentage of colonies formation following IAA treatment relative to their own control (NT). Each dot represents a single colony assay experiment. Error bars represent the SEM of four independent experiments. Unpaired t test: *** $p = 0.0003$. (**f**) Cell proliferation assay for up to 6 days in the indicated cell lines. Bioreduction of WST-1 into Formazan was measured at OD450nm. Error bars represent the SEM of a triplicate experiment. Unpaired t test: * $p = 0.0304$. (**g**) Points shows the measurement of acetyl-tubulin (in um) at mitotic exit in the indicated cell lines. Error bars represent the SEM of three independent experiments. Unpaired t test: ns. A representative image is also shown. Yellow arrow shows acetyl-tubulin localization. Scale bar = 5um. Source data for the immunoblot shown in (a) are provided as a Source Data file.

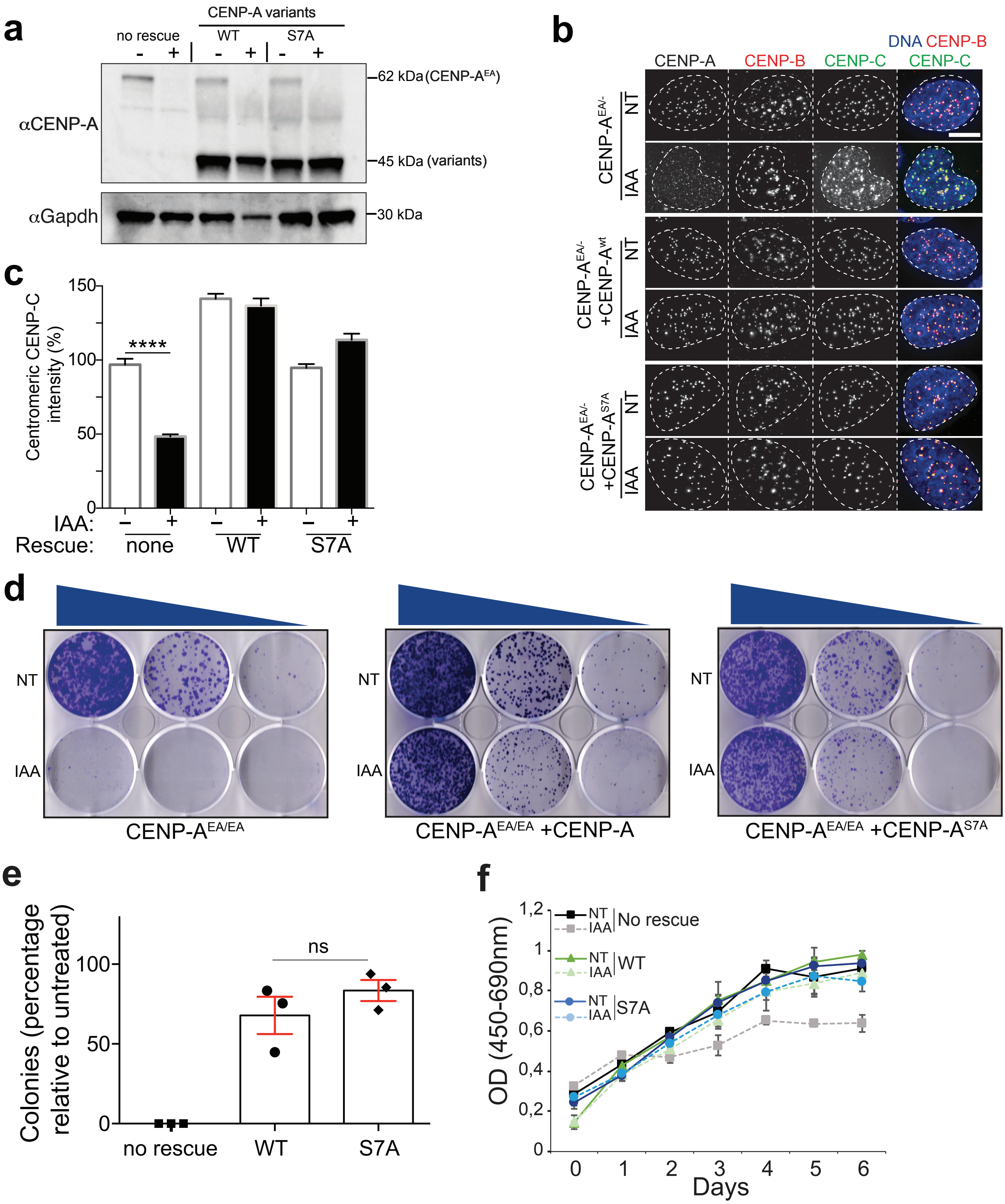

Supplementary Figure 2. CENP-A phosphorylation on serine 7 is not required for short term centromere function in HeLa cells (**a**) Immunoblots of cell extracts with antibody against CENP-A to determine the expression levels of exogenous CENP-A constructs, and the absence of endogenous CENP-A protein following IAA treatment. Antibodies against GAPDH are used as a loading control. N=2. (**b**) Representative immunofluorescence images showing the localization of exogenous CENP-A constructs in the indicated HeLa cell lines. CENP-C staining is also shown revealing its localization in presence of exogenous CENP-A constructs (IAA treatment for 24 hours). Scale bar = 5um. (**c**) Quantification of CENP-C levels of the experiments in panel b. Error bars represent the SEM of three independent experiments. Unpaired t test: **** $p < 0.0001$ (**d**) Representative images of crystal violet-stained colonies from the colony formation assay. N=3. (**e**) Bar graph shows the percentage of colonies formation following IAA treatment relative to their own control (NT). Each dot represents a single colony assay experiment. Error bars represent the SEM of three independent experiments. Unpaired t test: ns. (**f**) Cell proliferation assay for up to 6 days in the indicated cell lines. Error bars represent the SEM of a triplicate experiment. Bioreduction of WST-1 into Formazan was measured at OD450nm. Source data for the immunoblot shown in (a) are provided as a Source Data file.

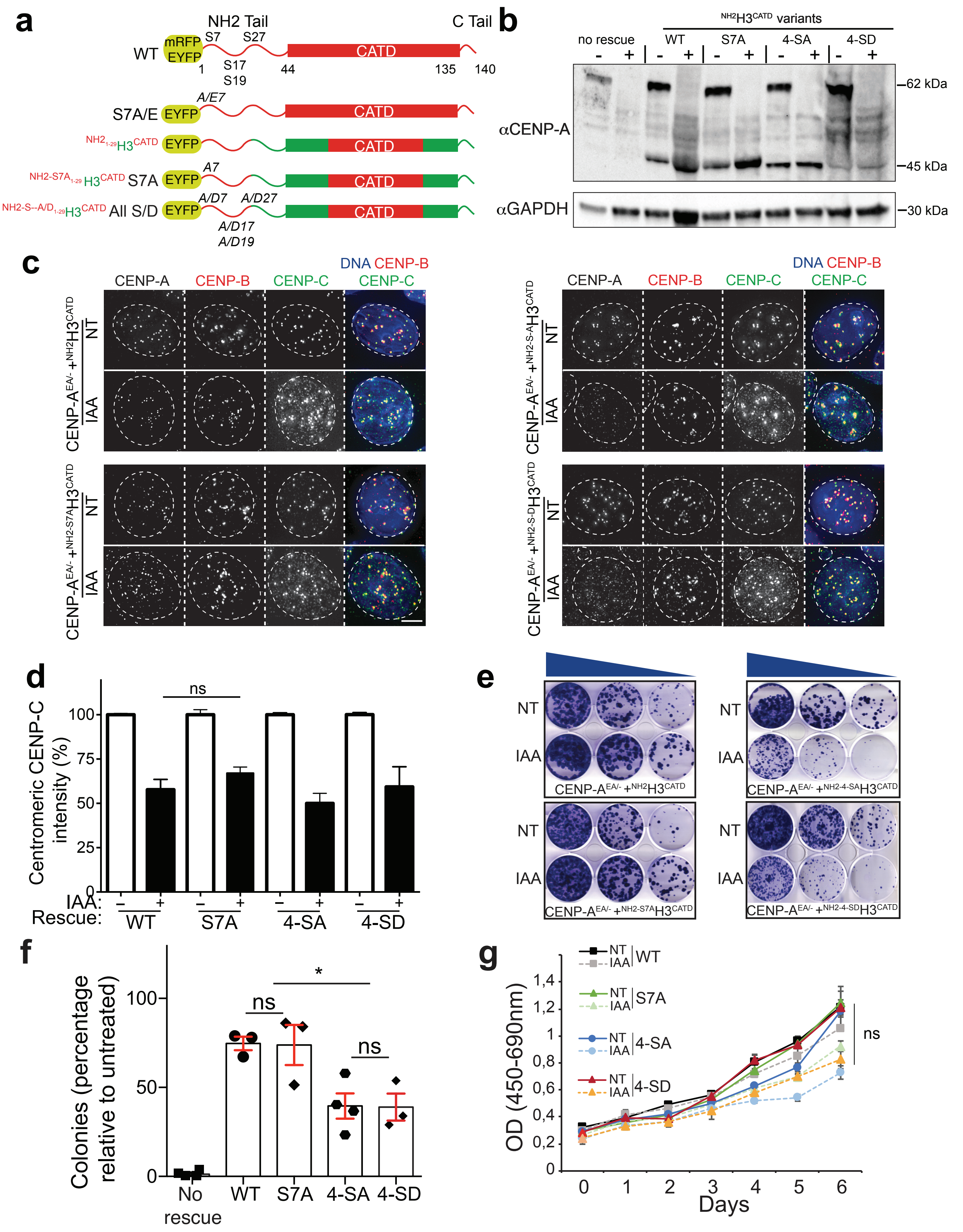

Supplementary Figure 3. Impairment of all CENP-A N-terminus phospho sites affects cell viability (**a**) Schematics representing the different CENP-A constructs amino-terminally tagged with EYFP. (**b**) Immunoblots of cell extracts with antibodies against CENP-A to determine the expression levels of exogenous constructs, and the absence of endogenous CENP-A protein in the IAA–treated cells. Antibodies against GAPDH are used as a loading control. (**c**) Representative immunofluorescence images showing the localization of exogenous CENP-A constructs in the indicated cell lines. CENP-C staining is also shown revealing its localization in the presence of exogenous CENP-A constructs (IAA treatment for 24 hours). Scale bar = 5um. (**d**) Quantification of CENP-C levels of the experiments in panel c. Error bars represent the SEM of three independent experiments. (**e**) Representative images of crystal violet-stained colonies from the colony formation assay. Cells were grown for 14 days to test their clonogenic survival in presence or absence of IAA. (**f**) Bar graph shows the percentage of colonies formation following IAA treatment relative to their own control (NT). Each dot represents a single colony assay experiment. Error bars represent the SEM of two/three independent experiments. Unpaired t test: * p = 0.028; ** p = 0.0029. (**g**) Cell proliferation assay for up to 6 days in the indicated cell lines. Bioreduction of WST-1 into Formazan was measured at OD450nm. Error bars represent the SEM of a triplicate experiment. Unpaired t test: ns. Source data for the immunoblot shown in (b) are provided as a Source Data file.

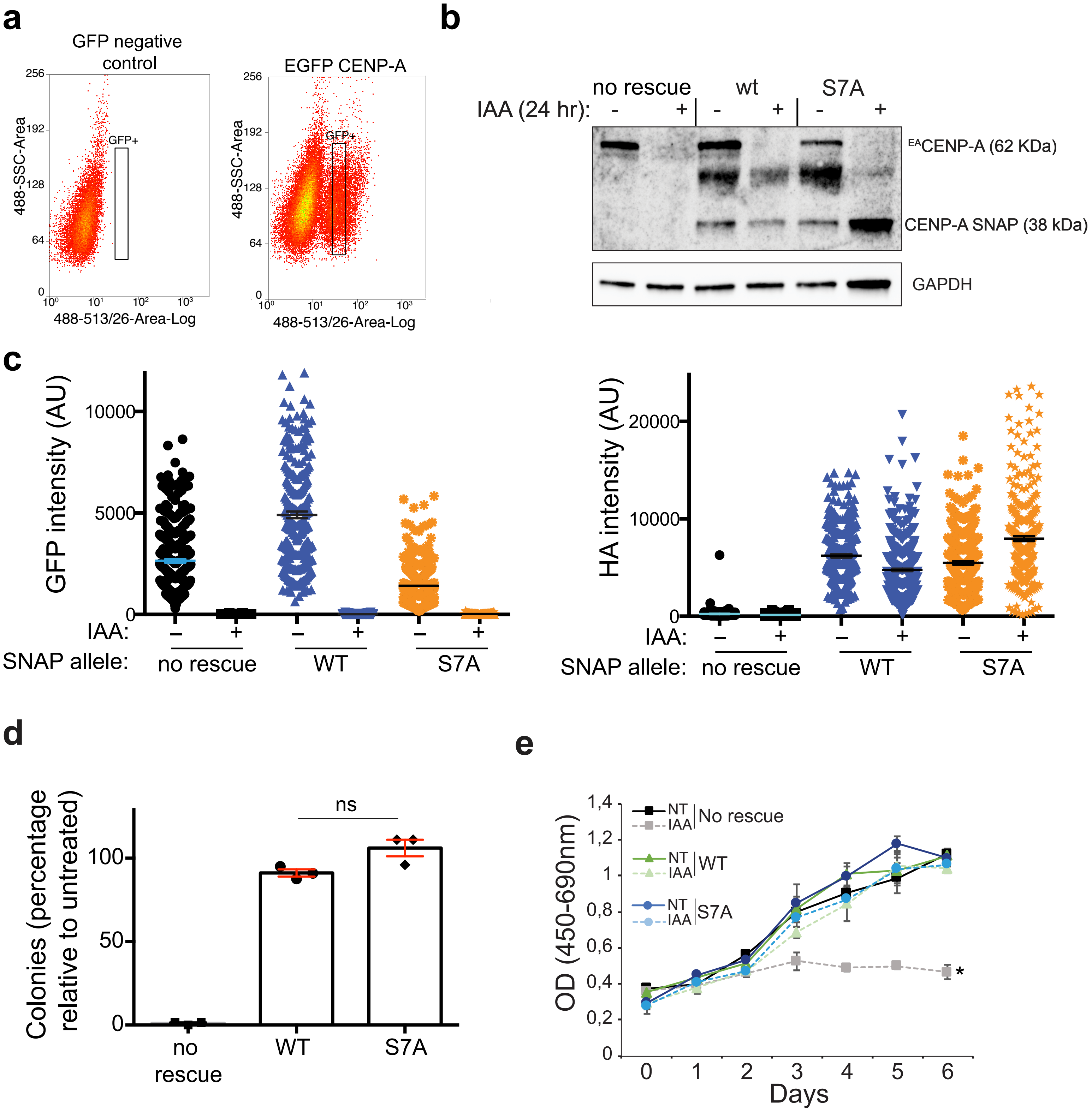

Supplementary Figure 4. Endogenous CENP-A un-phosphorylated serine 7 does not affect centromere maintenance and function (**a**) FACS plot shows the selection procedure of cells in which one CENP-A allele was targeted with EGFP-AID-CENP-A. (**b**) Immunoblots of cell extracts with antibodies against CENP-A to determine the expression levels of the two forms of CENP-A from both endogenous alleles. Antibodies against GAPDH are used as a loading control. N=2. (**c**) Quantification of CENP-A level at centromeric regions from cells expressing each allele (GFP tagged or HA tagged). (**d**) Bar graph shows the percentage of colonies formation following IAA treatment relative to their own control (NT). Each dot represents a single colony assay experiment. Error bars represent the SEM of three independent experiments. ANOVA test: * p = 0.01. (**e**) Cell proliferation assay for up to 6 days in the indicated cell lines. Bioreduction of WST-1 into Formazan was measured at OD450nm. Error bars represent the SEM of a triplicate experiment. Unpaired t test: * p = 0.029. Source data for the immunoblot shown in (a) are provided as a Source Data file.

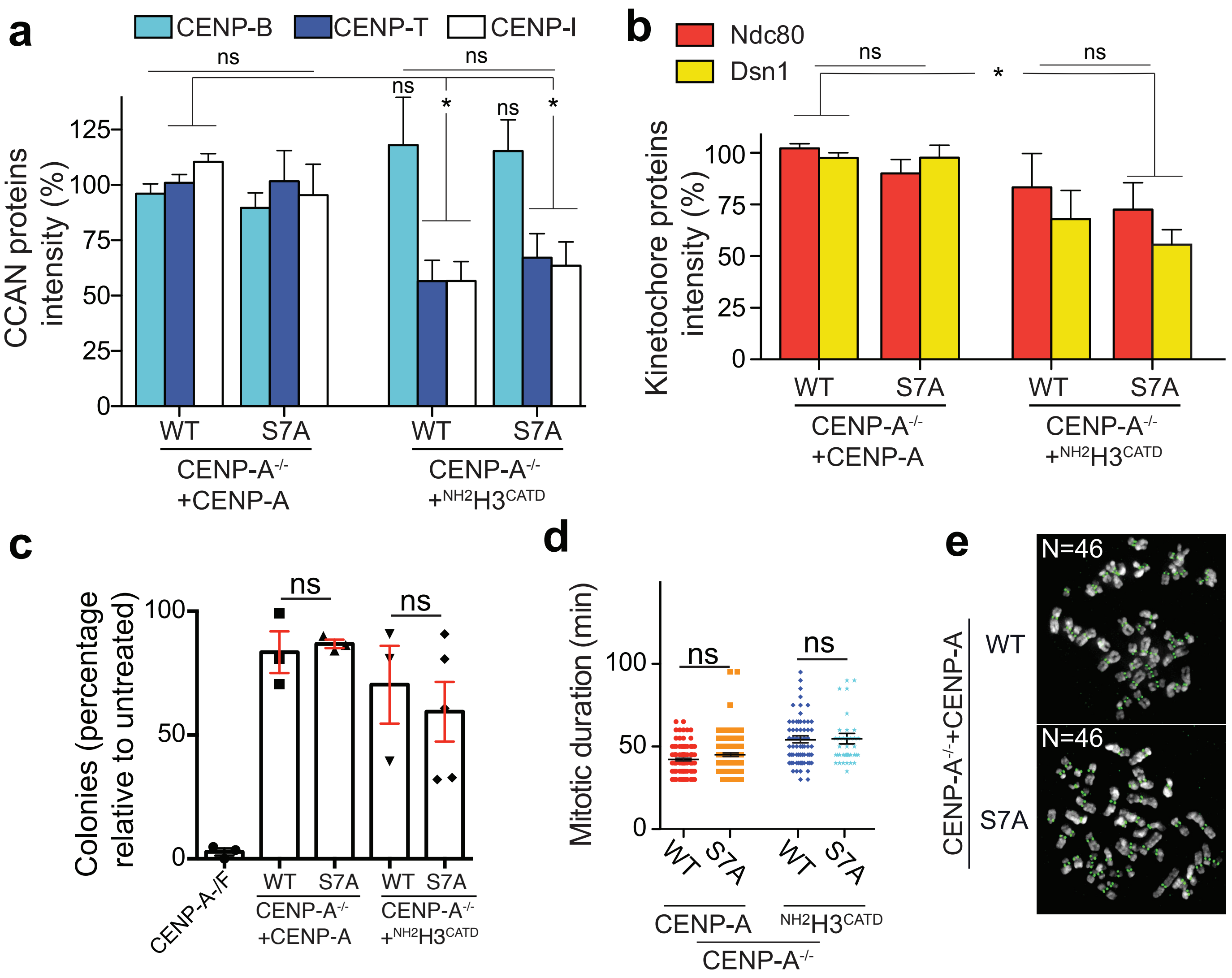

Supplementary Figure 5. CENP-A phosphorylation on serine 7 is not required for long-term centromere function (**a**,**b**) Quantification of CENP-B, CENP-T, CENP-I (CCAN proteins) and Ndc80 and Dsn1 (kinetochore proteins) in the indicated cell lines. Error bars represent the SEM of three independent experiments. ANOVA test: * p = 0.025; 0.036. (**c**) Bar graph shows the percentage of colonies formation following IAA treatment relative to their own control (NT). Each dot represents a single colony assay experiment. Error bars represent the SEM of three independent experiments. Unpaired t test: ns. (**d**) Quantification of mitosis duration in the indicated cell lines. Each individual point represents a single cell. Time in mitosis was defined as the period from NEBD to chromosome decondensation. Error bars represent the SEM of three independent experiments. Unpaired t test: ns. (**e**) Representative immunofluorescence images of metaphase chromosomes stained with CENP-A in the indicated cell lines. The number of chromosomes is also reported.

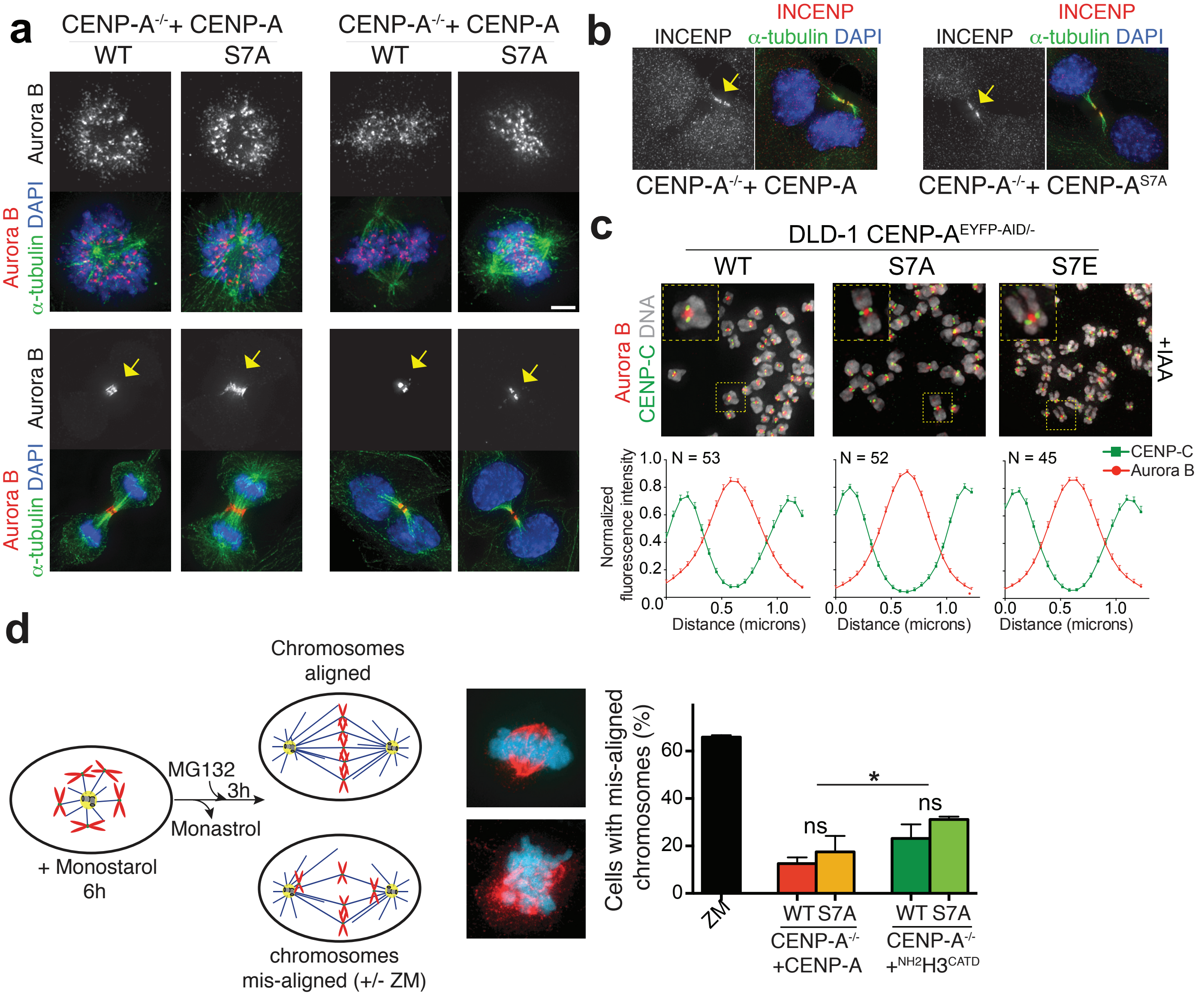

Supplementary Figure 6. CENP-A phosphorylation on serine 7 is not required for Aurora B localization and function (**a**) Representative immunofluorescence images showing the localization of Aurora B in prophase, metaphase, anaphase and telophase in the indicated cell lines. Scale bar = 5um. A yellow arrow marks Aurora B localization in anaphase and telophase. (**b**) Representative immunofluorescence images showing INCENP localization (marked by a yellow arrow) in telophase in the indicated cell lines. (**c**) Representative immunofluorescence images showing Aurora B staining in prometaphase-arrested cells. Line plots represent Aurora B (red) and CENP-C (green) intensities over localization in IAA conditions in the indicated cell lines. (**d**) (Left) Schematics of the experiment with monastrol treatment and release to test Aurora B function. Representative images of correct or incorrect chromosome alignment are shown. (Right) Quantifications of cells showing incorrect misalignment are shown. Error bars represent the SEM of three independent experiments. Unpaired t test: * p = 0.01.

a

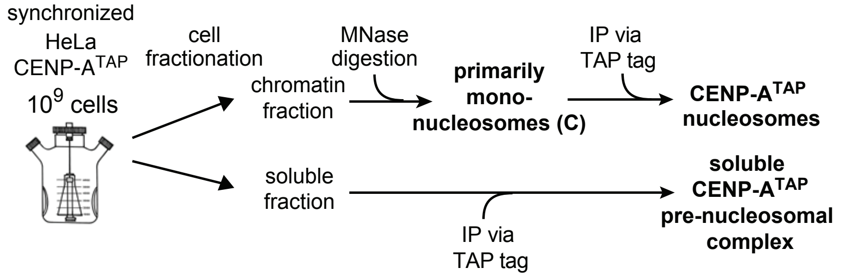

b

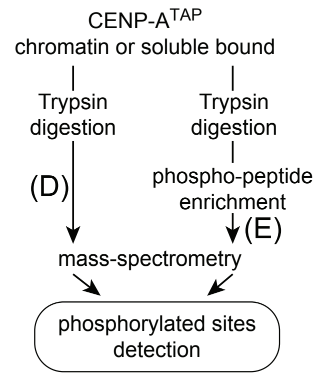

c

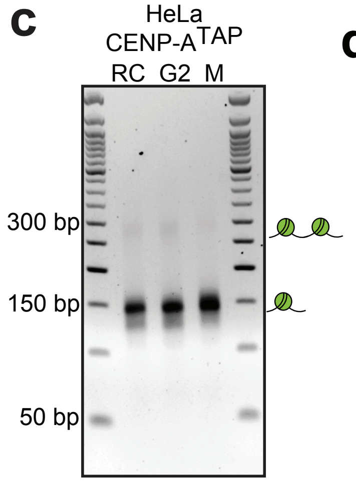

d

CENP-A chromatin-bound							
cell cycle phase	N° of peptides	Spectrum count	sequence coverage	peptides	identified phosphorylation	Ascore	occurrence
G1	3	95	22.10%	R.SPSPTPTPGPSR.R<br>K.STHLLIR.K<br>R.VTLFPKDVQLAR.R	SPS*PTPTPGPSR (S18)	32.32	7
mid S	2	9	17.10%	R.SPSPTPTPGPSR.R<br>R.VTLFPKDVQLAR.R	SPS*PTPTPGPSR (S18)	18.29	1
G2	4	573	29.30%	R.SPSPTPTPGPSR.R<br>K.STHLLIR.K<br>D.AYLLTLHAGR.V<br>R.VTLFPKDVQLAR.R	SPS*PTPTPGPSR (S18)	33.68	2

CENP-A soluble-bound				
cell cycle phase	peptides	Spectrum count	sequence coverage	peptides
G1	3	38	22.10%	R.SPSPTPTPGPSR.R<br>K.STHLLIR.K<br>R.VTLFPKDVQLAR.R
mid S	3	39	22.10%	R.SPSPTPTPGPSR.R<br>K.STHLLIR.K<br>R.VTLFPKDVQLAR.R
G2	3	87	22.10%	R.SPSPTPTPGPSR.R<br>K.STHLLIR.K<br>R.VTLFPKDVQLAR.R

e

Phospho-peptide enrichment

CENP-A chromatin bound			
	peptide	position	AScore
RC	RRS*PS*PTPTPGPSR	S16/S18	66.55 / 28.74
G2	RRS*PS*PTPTPGPSR	S16/S18	46.63 / 21.08<br>35.87 / 26.42
M	RRS*PS*PTPTPGPSR	S16/S18	57.39 / 24.81<br>47.69 / 24.81<br>45.02 / 34.98

CENP-A soluble bound			
	peptide	position	AScore
RC	RRS*PS*PTPTPGPSR<br><br><br>S*PS*PTPTPGPSR	S16/S18<br><br><br>S16/S18	66.81 / 39.78<br>40.07 / 18.34<br>27.57 / 27.99<br>58.33 / 35.92
G2	RRS*PS*PTPTPGPSR<br><br>RRS*PSPTPTPGPSR<br>S*PS*PTPTPGPSR	S16/S18<br><br>S16<br>S16/S18	66.55 / 34.28<br>57.39 / 31.51<br>44.78<br>58.33 / 33.7
M	none		

Supplementary Figure 7. Mass spectrometry analysis does not identify phosphorylation on CENP-A S7 (**a**) Schematics of CENP-A immunoprecipitations using the TAP tag from both chromatin and soluble fractions. (**b**) Schematics of mass spectrometry experiments to reveal CENP-A phosphorylation sites with or without the phosho-peptide enrichment. (**c**) Bulk chromatin from randomly cycling (RC), G2- and M-enriched cells was digested by MNase to produce a mono-nucleosome pool. DNA was extracted from the pool and ran on a DNA agarose gel. (**d-e**) Results of mass spectrometry following trypsin digestion ± phospho-peptide enrichment step (in e) from RC, G2 and M synchronized cells. AScore is a modification localization score. A value >13 indicates 95% confidence and a value of >20 indicates 99% confidence. *Asterisks denote phosphorylation sites.
