# Supplementary material for: Phosphorylation of CENP-A on serine 7 does not control centromere function
Source: Nat Commun. 2019 Jan 11;10:175. doi: 10.1038/s41467-018-08073-1 (PMC6329807; doi:10.1038/s41467-018-08073-1)
Supplement: Supplementary file 3 — Description of Additional Supplementary Files [file 41467_2018_8073_MOESM3_ESM.pdf]

Description of Additional Supplementary Files

Supplementary Data 1. Mass spectrometry analysis for PTM does not identify phosphorylation on CENPd A S7 (excel format)
