# Supplementary material for: Phosphorylation of CENP-A on serine 7 does not control centromere function
Source: Nat Commun. 2019 Jan 11;10:175. doi: 10.1038/s41467-018-08073-1 (PMC6329807; doi:10.1038/s41467-018-08073-1)

Figure 3b

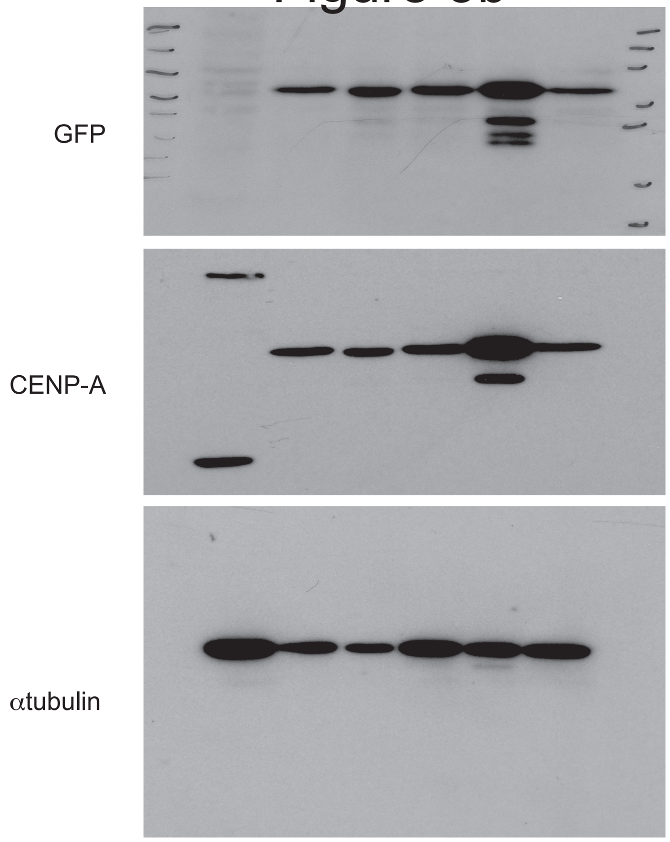

Supplemantary figure 1a

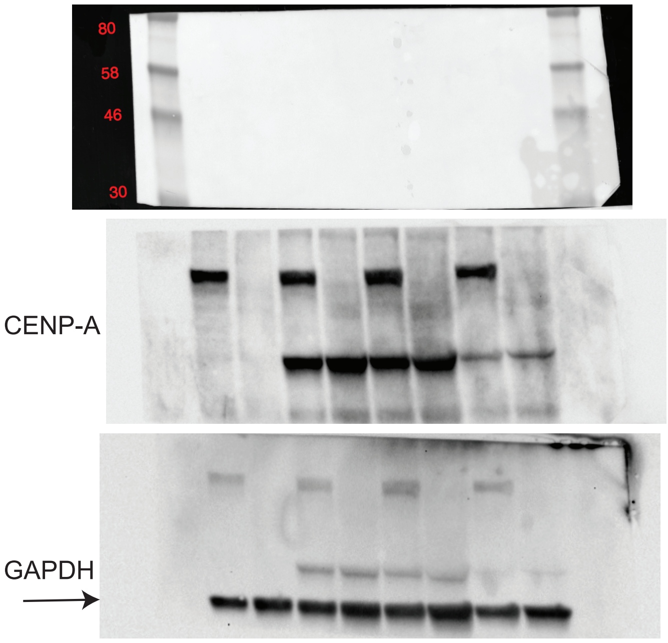

Supplemantary figure 2a

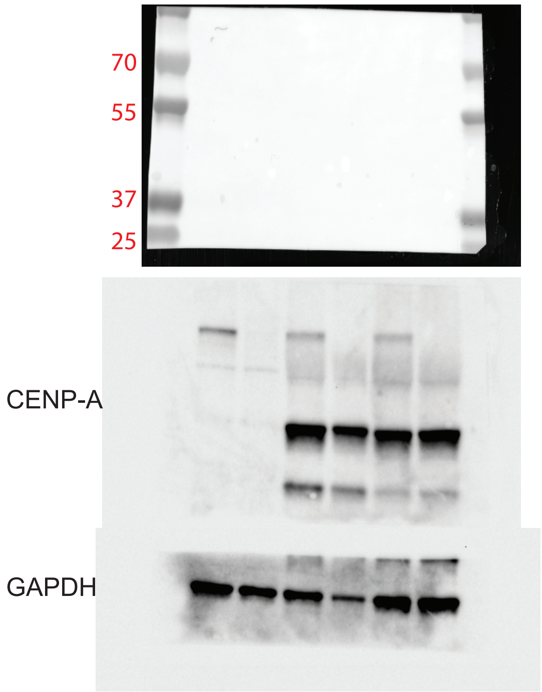

Supplemantary figure 3b

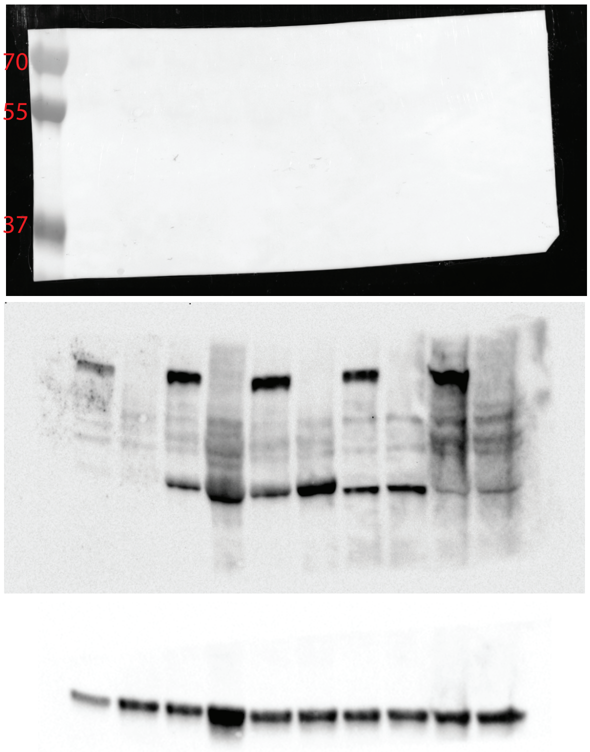

Supplemantary figure 4a

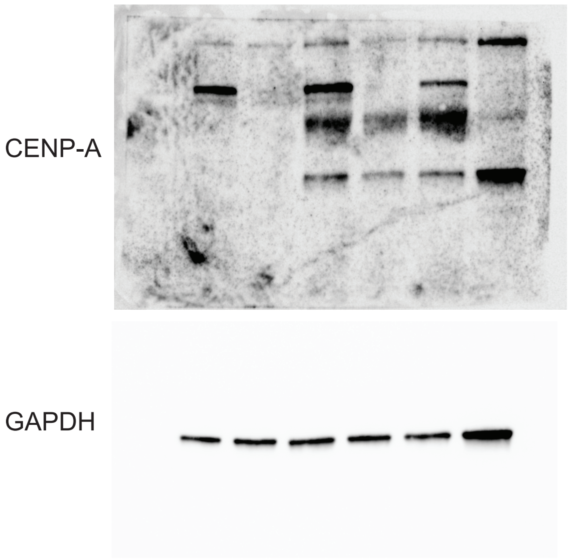

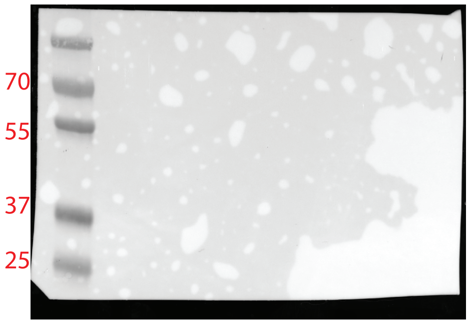

Supplement: Supplementary file 5 — Source Data File [file 41467_2018_8073_MOESM5_ESM.zip › Source Data blots.pdf]
